# Two year result of intravitreal bevacizumab for diabetic macular edema using treat and extend protocol

**DOI:** 10.1097/MD.0000000000006406

**Published:** 2017-04-21

**Authors:** Masahiko Sugimoto, Atsushi Ichio, Takayasu Nunome, Mineo Kondo

**Affiliations:** Department of Ophthalmology, Mie University Graduate School of Medicine, Tsu, Mie, Japan.

**Keywords:** bevacizumab, diabetic macular edema, treat and extend protocol

## Abstract

To determine the efficacy of the treat and extend (TAE) protocol with intravitreal bevacizumab (IVB) for managing diabetic macular edema (DME).

Retrospective, single-center study.

For this retrospective study, 42 eyes of 42 patients were initially treated with 3 consecutive monthly IVB injections (loading phase), after which they were selected for different additional therapies. For the TAE protocol, the baseline treatment interval was selected to be 8 weeks and was sequentially lengthened by 2 weeks if the central macular thickness (CMT) was <300 μm at 2 consecutive examinations.

Among the 42 eyes, 8 eyes (19.0%) received the TAE treatment for 2 years. The BCVA was improved significantly from 0.37 ± 0.04 before treatment to 0.19 ± 0.04 logMAR units at 2 years after the TAE determined IVB injections (*P* < .05). The ratio of eyes with a gain of the BCVA by more than 2 lines was 37.5%. The CMT was significantly reduced from 515.4 ± 75.5 to 303.6 ± 45.0 μm after 2 years (*P* < .01). The mean number of TAE injection was 8.8 and the mean injection interval was 11.0 weeks.

After the loading phase, 19.0% of patients can be treated with the TAE protocol. Although significant visual improvements were obtained after the TAE protocol, it does not apply to every DME case.

## 1 Introduction

Diabetic macular edema (DME) is a common cause of vision reduction in diabetic patients.^[1,2]^ Vascular endothelial growth factor (VEGF) has been shown to play a major role in the vascular proliferation and hyperpermeability in eyes with DME. Various anti-VEGF agents, bevacizumab (Avastin, Genentech), ranibizumab (Lucentis, Genentech), and aflibercept (Eylea, Regeneron Pharmaceuticals) are used to treat DME. The results showed that the anti-VEGF agents led to resolution of the macular edema (ME), and they have become the 1st-line therapy for DME.^[3–6]^ Although injection numbers decrease during years, their effectiveness is not a result of single injection, and repeat injections are required for many cases. The standard treatment protocol requires monthly clinic visits and injections which reduces the compliance and increases the cost of the treatments. Although a protocol that optimizes the risk-benefit balance has not been developed, a number of flexible strategies are being used.

Treatment as-needed or pro re nata (PRN) treatment is one of these strategies where patients receive fewer injections, and the timing of the injection is determined by a recurrence of the ME as assessed mainly by optical coherence tomography (OCT).^[7,8]^ Many physicians strive to reduce the number of injections and patient visits which would then reduce the stress and financial cost.

Much attention has been recently focused on a new protocol, called the treat and extend (TAE) protocol, for managing age-related macular degeneration (AMD), and many studies report that it can maintain a dry macular with reducing patient visits.^[9–13]^ TAE differs from the PRN protocol by determining the treatment interval by the condition of the ME as determined by clinical examinations. Thus, an individualized treatment and follow-up schedule is made for each patient.

We have reported on the effectiveness intravitreal bevacizumab (IVB) given as 3 consecutive monthly injections, loading phase, on the outcome at 5 months.^[14]^ We showed that 74% of the patient required further treatment including those being treated by the PRN or the TAE protocol. But the results were the short-term findings and longer postoperative findings are needed.

Thus, the purpose of this prospective study was to evaluate the visual and anatomical outcomes of IVB using a TAE protocol on eyes with DME.

## 2 Methods

This was a retrospective, single-center study of 42 eyes of 42 consecutive patients. Their mean age was 63.8 ± 11.6 years, and all were examined between May 2012 and August 2015 in the Department of Ophthalmology of the Mie University Hospital.

The procedures to be used and the possible complications were explained to the patients. In addition, the off-label use of bevacizumab was explained to all patients. A signed informed consent was obtained from all patients. Because no other anti-VEGF drugs including ranibizumab and aflibercept were approved before 2014 in Japan, bevacizumab was the only drug that could be used. The consent form also included a statement that the medical findings could be used for future research. The procedures used in this study were approved by the Institutional Ethics Review Board of the Mie University Hospital (#702), and they adhered to the tenets of the Declaration of Helsinki. List of the board's names are; Yoshiki Sugimura, Akihiro Sudo, Masaaki Narita, Norikazu Yamada, Kaname Nakatani, Yugo Narita, Yumi Eto, Kentaro Itagaki, Hironori Kawahara, and Tomoki Tamaru.

Each patient had a comprehensive ophthalmological examination including measurements of the best-corrected visual acuity (BCVA) and intraocular pressures, examination of the anterior segment by slit-lamp biomicroscopy, examination of the fundus by indirect ophthalmoscopy, and macular evaluations by spectral-domain OCT.

The inclusion criteria were: presence of DME, age at least 20 years, and BCVA pretreatment of 20/320 or better. The diagnosis of DME was determined by the clinical findings, fluorescein angiography, and a central macular thickness (CMT) greater than 300 μm in the spectral-domain OCT images. The exclusion criteria were: prior ocular surgery within 6 months, macular laser photocoagulation, and intravitreal or subtenon injections of steroid within 3 months before the IVB. In addition, eyes with ocular inflammation, drusen, severe proliferative diabetic retinopathy, retinal hemorrhage which involved the intra- or subfoveal spaces, an epiretinal membrane, any history of pars plana vitrectomy, glaucoma, and media opacities that significantly affected the BCVA were excluded. Patients with uncontrolled systemic medical conditions or history of thromboembolic events were also excluded.

### 2.1 Intravitreal bevacizumab (IVB) injection

IVB was injected under local subconjunctival anesthesia. Each patient received 1.25 mg of bevacizumab intravitreally with a 30-gauge needle that was inserted 4 mm posterior to the corneal limbus under sterile conditions. All patients received topical levofloxacin hydrate, (1.5% Cravit ophthalmic solution) for 1 week after the injection.

All patients were given 3 consecutive monthly IVB injections (the loading phase) as previously described,^[15,16]^ and they continued the therapies with IVB with the PRN or the TAE protocol. Other eyes were treated with intravitreal triamcinolone acetonide (IVTA), vitrectomy, or no treatment. Patients whose CMT shows improvement at 1 months after the loading phase were defined as bevacizumab-responder, and they were allowed to choose the PRN or TAE treatment. If there is a recurrence at that time, they received PRN injection. TAE schedule starts at 2 months after the loading phase because baseline treatment interval was defined as 8w for TAE treatment. On the other hand, those patients who did not show any improvement of CMT or did not acquire satisfactory vision improvement after the loading phase were excluded from further IVB treatment. They were switched to other therapies including PRN treatment, steroid injections, or vitrectomy. The treating physicians determined a course of alternative treatment after consultation with the patient.

### 2.2 Modified-TAE protocol for diabetic macular edema

The follow-up examination intervals were determined according to a modified-TAE protocol. After the loading phase, the baseline treatment interval was selected to be 8 weeks. The interval between treatments was increased by 2 weeks if the CMT was <300 μm at 2 consecutive examinations. The interval between the treatments was reduced by 2 weeks if the CMT was >300 μm or increased more than 20% of the baseline value.

### 2.3 Measurement of best-corrected visual acuity (BCVA)

The BCVA was measured with a Landolt chart at every visit. The decimal BCVA was converted to the logarithm of the minimum angle of resolution (logMAR) units for the statistical analyses.

### 2.4 Optical coherence tomography (OCT)

The degree of DME was determined from the images recorded by a Heidelberg Spectralis OCT instrument (Heidelberg Engineering Inc, Heidelberg, Germany). For qualitative and quantitative analyses of the OCT images, the fast macula protocol was used to obtain the images with an automatic real time mean value of 9 which acquired 25 horizontal lines consisting of 1024 A-scans per line. The CMT was defined as the thickness between the internal limiting membrane and the retinal pigment epithelium at the fovea, and the value was automatically calculated from the center subfield of the macular thickness map using the bundled software. The type of the DME, cystic, sponge-like, serous, and combined was based on the shape of the OCT images.^[17]^

### 2.5 Outcome measures

The primary outcome measure was the change in the BCVA at the conclusion of the 2-year study. Secondary outcomes were the changes in the CMT and the number of injections given over the 2 years.

### 2.6 Statistical analyses

Statistical analyses were performed with the SPSS software package (SPSS Inc., Chicago, IL). The results are presented as the means ± standard deviations (SD). Two-way repeated measures ANOVA and post-hoc *t* tests with Bonferroni corrections were used to determine the significance of the changes in the BCVA and CMT. Two-tailed *P* values of <.05 were considered to be significant.

## 3 Results

**Figure 1:**
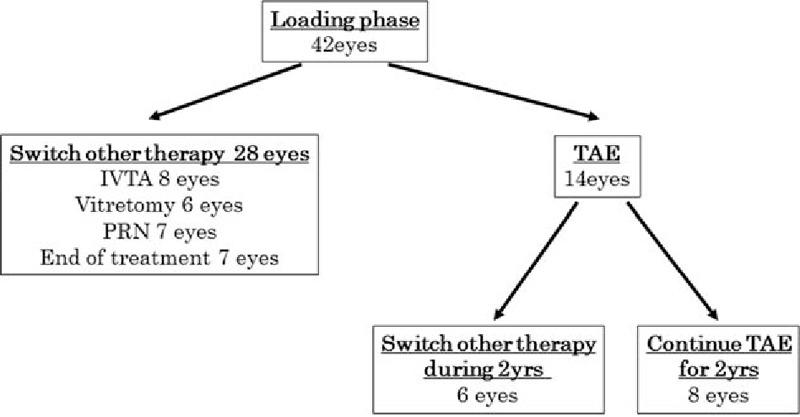
Flow chart showing progression of subjects through the study. IVB = intravitreal bevacizumab, IVTA = intravitreal triamcinolone acetonide, PRN = pro-re-nata, TAE = treat and extend.

Forty-two patients met the inclusion criteria, and each received the loading phase treatment of 3 continuous IVB injections (Fig. 1). After the loading phase, 14 eyes (33.3%) proceeded to the TAE treatment and 28 eyes were switched to other therapies. The other therapies included IVTA (8 eyes), vitrectomy (6 eyes), IVB with PRN protocol (7 eyes), and no treatment (7 eyes). The end of treatment indicates that these patients preferred no further treatment because adequate vision was not attained though the reduction of the edema was obtained during the loading phase. These patients can obtain anatomical improvement after loading phase but cannot obtain functional improvement. The PRN injection protocol was performed when the CMT was >350 μm, and the patients requested additional IVB injections. Among the 14 TAE patients, 6 eyes had to change treatment during the 2-year follow-up period (IVTA 3 eyes, virectomy 1 eye, and PRN 2 eyes). The mean baseline BCVA of these 6 eyes was 0.51 ± 0.33 logMAR units, and the mean baseline CMT was 525.2 ± 139.9 μm. The BCVA improved to 0.35 ± 0.20 logMAR units after the loading phase, and mean final BCVA at 2 years improved to 0.36 ± 0.18 logMAR units. The CMT of these 6 eyes also improved to 400.8 ± 120.0 after the loading phase, and the final mean CMT at 2-years was 421.2 ± 183.9 μm, but there was no significance during the observation for both BCVA and CMT.

Finally, 8 eyes (19.0%) received TAE treatment for 2 years. The clinical characteristics of these 8 eyes are shown in Table 1. There were 7 men and 1 woman whose mean age was 55.4 ± 10.9 (mean ± standard deviations) years in the 2 years treated TAE group. The mean baseline BCVA was 0.37 ± 0.04 logMAR units, and the mean baseline CMT was 515.4 ± 75.5. The BCVA improved to 0.30 ± 0.03 logMAR units after the loading phase, and mean final BCVA improved significantly to 0.19 ± 0.04 logMAR units *(P* < .05). The mean change in the BCVA was 0.18 ± 0.04 logMAR units, and the ratio of eyes with an improvement of the BCVA of more than 2 lines to the total number of eyes was 37.5% (3/8).

**Table 1:**
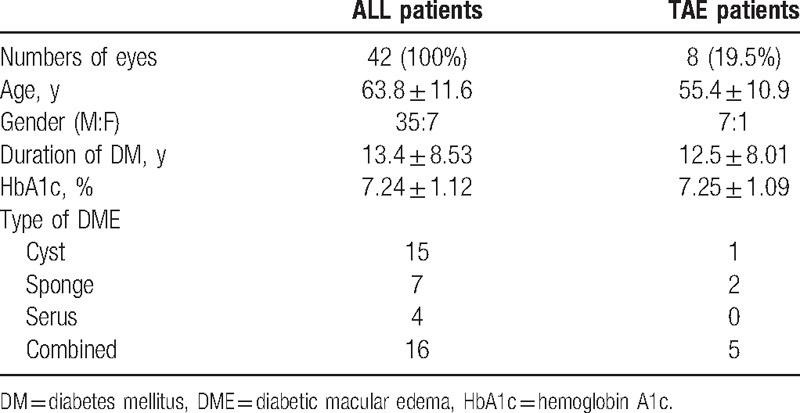
Demographics of TAE patients.

The CMT was significantly reduced from 515.4 ± 75.5 to 438.3 ± 64.0 μm after the loading phase, and the final mean CMT at 2-years was reduced significantly to 303.6 ± 45.0 μm (*P* < .05; Table 2).

**Table 2:**
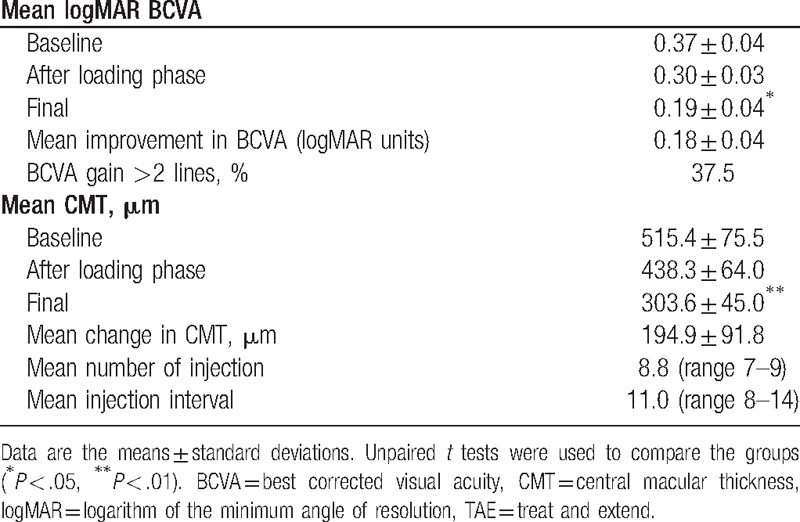
Outcome of 2-years TAE treatment (n = 8).

**Figure 2:**
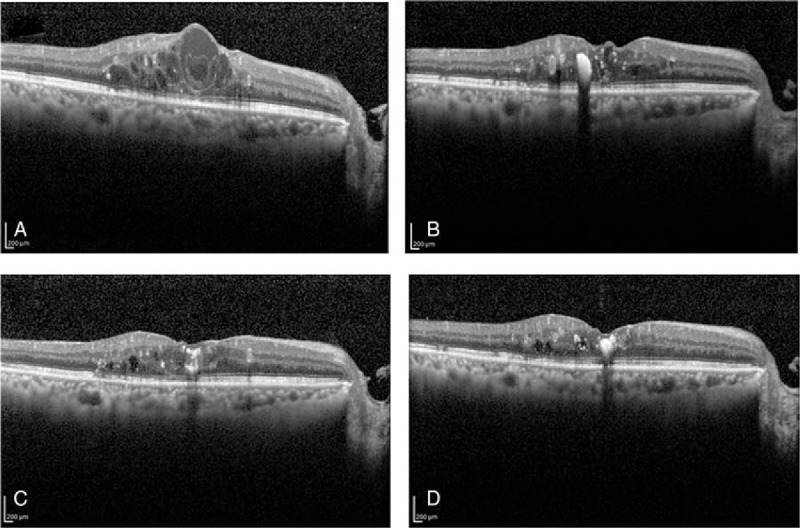
Representative case treated with the TAE protocol. Before treatment, his log MAR BCVA was 0.4 and the CMT was 632 μm (A). After the loading phase, cystic edema resolved and the log MAR BCVA was 0.4 and the CMT was 491 μm (B). Then, 4 IVB injections with the TAE protocol were performed, the log MAR BCVA was 0.22, and the CMT was reduced to 266 μm (C). After 10 injections of IVB with the TAE protocol, the log MAR BCVA was improved to be 0.15 and CMT also improved to 232 μm with a TAE interval of 12 weeks. BCVA = best corrected visual acuity, CMT = central macular thickness, IVB = intravitreal bevacizumab, TAE = treat and extend protocol.

The mean number of TAE injections was 8.8, and the mean injection interval was 11 weeks during the 2 years. There were no patients with a decrease of the visual acuity from baseline at the conclusion of the study. There were no IVB-related ocular complications including intraocular pressure elevation, infections, or episodes of systemic adverse events. A representative case of TAE treatment is shown in Fig. 2.

## 4 Discussion

Our results showed that IVB with the TAE protocol for 2 years resulted in significant visual and anatomical improvements in eyes with DME. The TAE protocol was effective in those patients who continued for at least 2 years. However, it was not effective in all cases, and not many patients were adapted for the TAE protocol (19.0% for 2 years). Thus, some of the patients had to switch to other therapies during the 2-year follow-up period.

The difficulty of implementing the PRN protocol is that it requires frequent examinations so that a recurrence can be detected early and treated before irreversible damage occurs. In addition, PRN-treated patients always fear the long-term vision prognosis including the risk of recurrences. TAE is a modified PRN treatment regimen that aims to maintain an exudation-free macula while simultaneously reducing the frequency of patient visits and diagnostic testing. This protocol was put forward as an “inject and extend” treatment for AMD.^[9]^ Once the maximal response, that is, anatomical improvement or stabilization of the VA, was obtained, the treatment intervals could be extended, but the patients still received an injection at every visit to protect against a recurrence of the ME. In contrast to conventional PRN, TAE subjects were treated at each visit which can reduce the episodes of recurrent exudations. The usefulness of the TAE protocol for AMD and the ME associated with branch retinal vein occlusion are well known.^[10–13,18,19]^ The TAE protocol can reduce frequency of visiting and has the potential of reducing stress of both patients and physicians while maintaining good visual and anatomic outcomes. The subjects in our study with the TAE protocol required fewer office visits (range 7–9 during 2 years) and had a greater stability of their vision. In addition, the increased cost because of the frequent visits with the PRN protocol can become a problem, and the fewer TAE monthly injection can reduce the cost.^[20,21]^

The RETAIN study reported their results of TAE treatment for DME.^[22]^ All of the patients received monthly ranibizumab injection until the VA stabilized, and then the visiting interval was incrementally increased by 1 month steps in the VA-stable patients. There was a reduction of approximately 40% in the examination visits with TAE, and approximately 70% of the TAE patients had a monitoring interval of ≥2 months. They concluded that the TAE regimen led to a reduction in the number of visits. Their findings are comparable with our results indicating the usefulness of TAE. However, it is difficult to make a more detailed comparison of their results with our results because the demographics of the patients and injection criteria were different. In addition, they used injection intervals of up to 3 months, and many injections and frequency of visiting were needed.

Overall, the TAE protocol for DME appears to be effective but there are some disadvantages; there is a possibility of over treatment, difficulty of identifying a stable status without treatment, increased chance of adverse complications, limited evidence, and no stop criteria.^[23]^ In addition, the number of injections is more than that with the PRN protocol, and schedule planning is difficult. The injection and extension criteria are simply for AMD, but there is no established protocol for DME. The criteria for an extension are also an issue; what is the best measure to determine the extension intervals, for example, a CMT of 300 or 350 μm. Strict criteria make it difficult to determine the extension interval, and it can become a bimonthly treatment protocol. On the other hand, a permissive criterion is not reasonable if the extension criteria is defined as, for example, a CMT <400 μm. In fact, our criterion was a CMT <300 μm, and we could not extend the interval between visits to over 8 weeks for 2 eyes, that is, bimonthly injections. Although we used the OCT findings for determining the extensions, the same as the AMD-TAE protocol, the BCVA-based protocol may better reflect the actual situation for DME as in the RETAIN study.^[22]^ Thus, there are some limitations in performing TAE for DME based on only the OCT findings. We believe it is important to develop a treatment regimen for each case based on the findings in the loading phase, which may be better than a fixed monthly or bimonthly injection schedule.

The 5 year results of the DRCR.net Protocol I study showed that it takes 4 to 5 years to reach a stage when the ranibizumab injections can be stopped,^[6]^ and it is still difficult to apply these results to patients because monthly or bimonthly injections cause a great burden on the patients. Three consecutive monthly injections as the loading phase injection is a protocol frequently used for AMD, and it is also used for DME treatments to avoid such problems.^[24]^ Although the effectiveness of anti-VEGF agents for DME is well known, it is important to know that we cannot treat all patients with only this therapy. The DRCR.net protocol I also showed that the CMT of 60% of the patients can be reduced to <250 μm which indicates that 40% of the patients had CMT >250 μm. These were non- or weak responder to anti-VEGF therapy.^[5]^ Its ratio is not so high, but there are still those whose vision will be severely reduced unless they continued anti-VEGF treatment.^[25]^ Three consecutive monthly injection as a loading phase is the proper number of injections to test for the nonresponders at an early stage of treatment up to 3 months. For nonresponders, we have to switch to other therapies including steroid therapy or vitrectomy once we decide that the anti-VEGF treatment is not effective to avoid outer segment damage induced by prolonged edema. In fact, many patients in our study switched to other therapy after the loading phase. After persistent edema caused irreversible damage to the outer segments of the photoreceptors, better visual function cannot be achieved even though the edema was resolved. We have various choices for treating DME, and it is possible to switch to other therapy after we determined how the patients respond to the initial set of injections.

Although our study showed the efficacy of TAE protocol, some problems remain. One major limitation of our study was the small number of eyes. On this point, we have to evaluate patients as a multicenter study. Second, though many patients preferred or switched to other therapy, the criteria are not objectively defined. However, in the real world, the physicians have to determine a course of alternative treatment after consultation with the patient. On this point, our result reflects practical treatment. Especially, criteria and protocol of PRN treatment was not strict. We performed PRN injections based on the symptoms of the patients and not only on the CMT but mainly on the BCVA. Although the criteria for determining the time for another IVB injection was not fixed, we compared the 2 year results with that of the TAE protocol. There was significant difference in the CMT (303.6 ± 45.0 μm for TAE vs 331.3 ± 21.0 for PRN at 2 years), and BCVA (0.19 ± 0.04 logMAR units for TAE vs 0.35 ± 0.09 logMAR units for PRN at 2 years; *P* < .01). This result lacks credibility because these differences are probably because we used strict criteria for TAE but not for PRN. In addition, we used bevacizumab which is not as expensive as ranibizumab or aflibercept. Patients may prefer PRN more if they mind such cost, so we have to establish definite criteria for the PRN and TAE protocols for the patients.

In conclusion, IVB with the TAE protocol for 2 years resulted in significant visual and anatomical improvements. Our results showed that TAE was effective for those patients who can continue 2 years of follow-up examinations but is not for all cases. Our findings indicate that it is important to establish strict criteria for using the TAE treatment protocol for eyes with DME.

## Acknowledgments

The authors thank Professor Emeritus Duco Hamasaki of the Bascom Palmer Eye Institute of the University of Miami for critical discussion and final manuscript revisions.
